# Indication of West Nile Virus (WNV) Lineage 2 Overwintering among Wild Birds in the Regions of Peloponnese and Western Greece

**DOI:** 10.3390/vetsci10110661

**Published:** 2023-11-18

**Authors:** Zoi Athanasakopoulou, Marina Sofia, Vassilis Skampardonis, Alexios Giannakopoulos, Periklis Birtsas, Konstantinos Tsolakos, Vassiliki Spyrou, Dimitris C. Chatzopoulos, Maria Satra, Vassilis Diamantopoulos, Spyridoula Mpellou, Dimitrios Galamatis, Vasileios G. Papatsiros, Charalambos Billinis

**Affiliations:** 1 Faculty of Veterinary Science, University of Thessaly, 43100 Karditsa, Greece; zathanas@uth.gr (Z.A.); msofia@uth.gr (M.S.); bskamp@uth.gr (V.S.); algiannak@uth.gr (A.G.); 2 Faculty of Forestry, Wood Science and Design, 43100 Karditsa, Greece; birtsas@uth.gr; 3 Hunting Federation of Peloponnese, 26221 Patra, Greece; kostastsolakos@hotmail.com; 4 Faculty of Animal Science, University of Thessaly, 41110 Larissa, Greece; vasilikispyrou@uth.gr (V.S.); dgalamatis@uth.gr (D.G.); 5 Faculty of Public and One Health, University of Thessaly, 43100 Karditsa, Greece; dchatzopoulos@uth.gr (D.C.C.); msatra@uth.gr (M.S.); 6 Directorate of Public Health, Prefecture of Peloponnese, 22132 Tripoli, Greece; diamantopoulos@ppel.gov.gr; 7 Bioefarmoges Eleftheriou LP-Integrated Mosquito Control, 19007 Marathon, Greece; smpellou@bioefarmoges.com; 8 Clinic of Medicine, Faculty of Veterinary Medicine, University of Thessaly, 43100 Karditsa, Greece; vpapatsiros@vet.uth.gr

**Keywords:** West Nile virus, wild birds, Greece

## Abstract

**Simple Summary:**

West Nile virus (WNV) is one of the most widespread zoonotic arboviruses worldwide and wild avian species act as its amplifying hosts in nature. In the present study, WNV circulation among wild birds was evaluated in two regions of Greece during 2022. A total of 511 birds were sampled and screened for WNV RNA. The virus was detected in 71 samples from both regions, during February to November. Population density and distance from water sources were identified as important factors associated with WNV occurrence. In conclusion, these findings show that WNV circulated in both investigated regions of Greece during 2022, highlighting the need for avian species surveillance to be conducted annually and throughout the year. Magpies are proposed as sentinels for WNV monitoring.

**Abstract:**

West Nile virus (WNV), a zoonotic mosquito-borne virus, has recently caused human outbreaks in Europe, including Greece. Its transmission cycle in nature includes wild birds as amplifying hosts and ornithophilic mosquito vectors. The aim of this study was to assess WNV circulation among wild birds from two regions of Greece, Peloponnese and Western Greece, during 2022. To this end, a total of 511 birds belonging to 37 different species were sampled and molecularly screened. WNV RNA was detected from February to November in a total of 71 wild birds of nine species originating from both investigated regions. The first eight positive samples were sequenced on a part of NS3 and, according to the phylogenetic analysis, they belonged to evolutionary lineage 2 and presented similarity to previous outbreak-causing Greek strains (Argolis 2017, Macedonia 2010 and 2012). It was more likely to identify a PCR positive bird as the population density and the distance from water sources decreased. The present report provides evidence of WNV occurrence in both Peloponnese and Western Greece during 2022 and underlines its possible overwintering, highlighting the need for avian species surveillance to be conducted annually and throughout the year. Magpies are proposed as sentinels for WNV monitoring.

## 1. Introduction

West Nile virus (WNV) is a zoonotic flavivirus that has evolved to be one of the most widespread arboviruses in the world [1]. It is transmitted via ornithophilic mosquitoes, mainly of the *Culex* genus, particularly to avian species but also to mammalian hosts [2]. WNV was first described in Uganda in 1937 [3]. Since then, sporadic cases and several outbreaks were reported in Africa, the Middle East, Europe and Asia [4]. Introduction of the virus in the United States was reported in 1999 and was characterized by significant avian morbidity (mainly *Corvidae* spp.) in the area of New York, followed by morbidity in humans. Over a decade, WNV has spread in all continents except Antarctica [5]. In the last few years, WNV cases have been observed particularly in Southern Europe, often associated with the major flyways of migratory birds [6]. Annual late-summer outbreaks of WNV occur regularly in European countries that border the Mediterranean Sea and the virus is endemic in some regions [7].

The host range for WNV is wide and includes over 300 species of birds, mammals, reptiles and amphibians [8,9]. Wild birds are considered the main reservoir of the virus, which is maintained in nature in an enzootic bird–mosquito cycle [10]. The replication of WNV varies among hosts and is significantly higher in birds compared to other species [8]. Some bird species may have high levels of viremia for an extended time without presenting clinical signs and therefore could contribute to the maintenance of the virus in nature as well as to its geographical expansion during annual migrations. Nevertheless, some species of raptors, jays and crows are highly susceptible, and can develop severe or fatal encephalitis [11]. Clinical infection in susceptible, incidental, dead-end hosts (humans and horses) is usually asymptomatic, but may also result in mild febrile disease with flu-like symptoms or occasionally in neuroinvasive disease, manifested with neurological deficits (ataxia, weakness and mental disturbances) and possibly death [12].

In Greece, the first report regarding the detection of WNV antibodies in wild birds (*Pica pica* and *Corvus cornix*) dates back to 2009 in Central Macedonia [13]. Seropositive birds (*Pica pica*, *Corvus cornix* and *Streptopelia turtur*) were also identified in the same area during 2010–2011, while molecular detection of WNV lineage 2 was performed from a magpie near the village Trilofos in 2010 [13,14]. Positive avian sera (*Pica pica* and *Streptopelia turtur*) were additionally reported in 2011 in the regional units of Serres, Thessaloniki, Trikala, Larissa and Karditsa [15]. In 2017, Eurasian magpies with neurologic signs were identified in the Argolida regional unit of the Peloponnese region and subsequently WNV was molecularly detected [16]. Recently (2020), the presence of the virus was reported in ten different wild bird species (*Pica pica, Passer domesticus, Parus major, Passer hispaniolensis, Garrulus glandarius, Corvus monedula, Ardea purpurea, Athene noctua, Strix aluco* and *Curruca communis*) originating from Peloponnese [17]. Human WNV infections as well as deaths were initially reported in the country in 2010 and by 2021 a total of 1420 cases including 201 fatalities were recorded from different regions [18]. 

WNV lineages 1 and 2 are the most widely spread and the most virulent of the virus’ lineages, capable of causing numerous cases worldwide [19]. WNV lineage 2 was first detected in Europe in 2004, with its isolation from the brain of a goshawk (*Accipiter gentiles*) in Hungary [20]. Subsequently, it emerged in various countries including Greece, Italy, Romania and Serbia, and it is now regarded as an important established problem around the world with a high risk for further spread, particularly in countries around the Mediterranean Sea [7]. Notably, WNV lineage 2 in Greece was responsible for a severe outbreak in 2010, causing numerous human cases with neurological manifestations and deaths [21,22], and it continues to be associated with several human cases each year [23,24]. 

Considering the established presence of WNV in Greece and the acknowledged value of wild bird surveillance systems in evaluating virus’ circulation and supporting vector control decisions [7,25], the present study aimed to assess the occurrence of WNV in a sample of birds from two neighboring regions of Greece, namely, Peloponnese and Western Greece, during the year 2022. In addition, in order to evaluate similarity with previous circulating strains, a phylogenetic analysis was conducted, while the association of environmental parameters with the presence or absence of WNV in wild birds was also investigated. 

## 2. Materials and Methods

### 2.1. WNV Surveillance in Greece

In Greece, WNV surveillance of all the laboratory-diagnosed human cases of WNV infection is supervised by the National Public Health Organization (NPHO). All probable and confirmed cases are investigated within 24 h after diagnosis and there is a daily follow-up of all hospitalized cases until discharge. Affected areas are defined as third administrative level units with at least one human case [26].

The Greek Ministry of Rural Development and Food implements surveillance strategies among the non-human vertebrate hosts, and in particular among equids and wild birds [27]. Regarding equids, the program includes (i) active serological surveillance of sentinel horses, (ii) active clinical surveillance of equids around confirmed human or/and animal cases and (iii) passive surveillance of WNV fever in equids all year round. Concerning wild birds, (i) passive surveillance is implemented by sampling dead or sick wild birds throughout the country and (ii) active surveillance is conducted in a finite number of samples at selected areas of eight regional units, mainly near lakes and rivers [26,27]. In addition, the Laboratory of Microbiology and Parasitology, Faculty of Veterinary Science, University of Thessaly and the Ministry of Rural Development and Food have concluded a Memorandum of Cooperation for WNV surveillance in wild bird species. Specimens from susceptible dead birds as well as from living ones of high-risk areas are examined for virus presence throughout the year.

### 2.2. Study Area and Collection of Biological Material from Wild Birds

**Figure 1:**
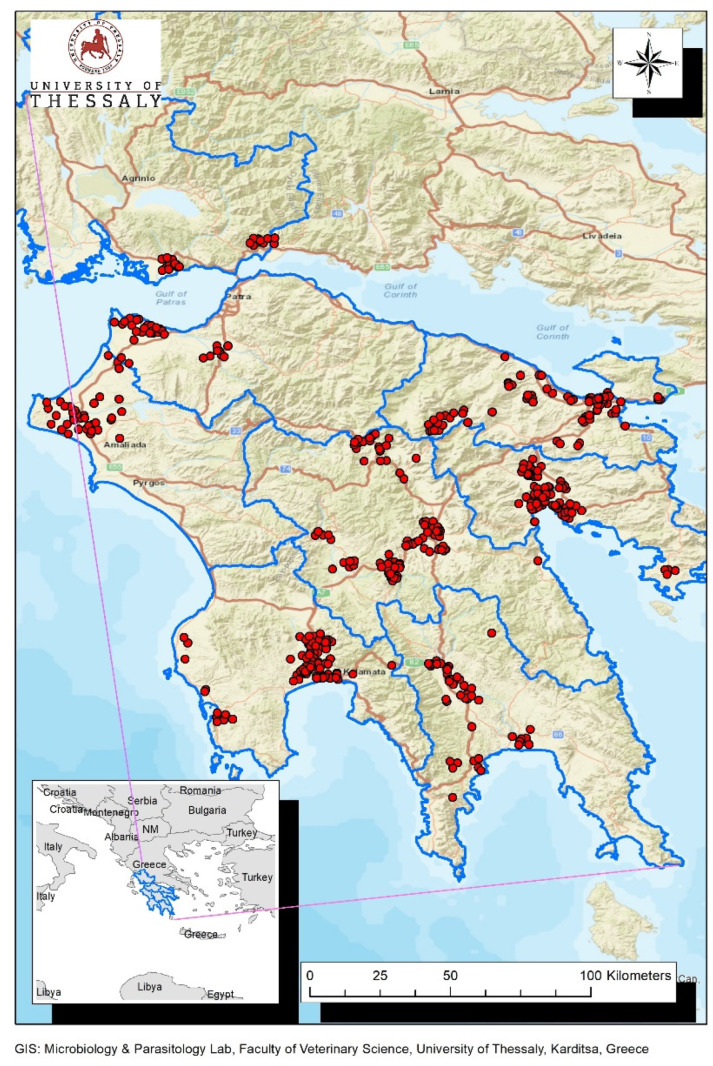
Map of Peloponnese and Western Greece regions. Sampling sites of wild birds are depicted with red dots. The boundaries of the regional units (Peloponnese region: Argolis, Arcadia, Laconia, Corinthia and Messenia, Western Greece region: Achaia, Ilia and Aitoloakarnania) are outlined with blue color.

The present study was conducted in two distinct neighboring administrative entities of Greece, the region of Peloponnese and the region of Western Greece (Figure 1). The region of Peloponnese includes the regional units Argolis, Arcadia, Laconia, Corinthia and Messenia, while the region of Western Greece comprises the regional units Achaia, Ilia and Aitoloakarnania. 

In both regions, observations of wild bird species were conducted during 2022 regarding resident species that could be a WNV reservoir and migratory species that could contribute to the introduction of new virus strains. Point-count stations, line-transects and direct count stations were used as recording techniques for their monitoring. 

Sampling of wild birds was performed in diverse ecological niches of natural ecosystems, suburban and urban areas. Small cage traps (20 cm × 30 cm × 20 cm), Larsen Traps (60 cm × 50 cm × 50 cm), Multi Catch Larsen Traps (160 cm × 200 cm × 150 cm), groundnets and mistnets were used for capturing. To maximize the number of collection sites, portable traps were moved periodically to different locations and habitat types. The sampling procedure included collection of blood samples, and/or oropharyngeal swabs and/or fecal swabs (Copan). Upon the end of specimen collection, birds were released into their natural habitats according to the prerequisites of the Greek Legislation. Furthermore, environmental fecal samples were obtained by inserting a sterile cotton swab into recently deposited feces (n = 21). Fresh bird carcasses (n = 18) found alongside the road network during the field work were also collected. Tissue samples (i.e., brain, heart, liver, spleen and kidney) were acquired by performing necropsies and stored immediately at −70 °C until molecular screening. During the 2022 official hunting season, hunter-harvested tissue samples (n = 472) were additionally shipped to the laboratory from the Greek Hunting Federation of the Peloponnese region. Coordinates of all sampling sites were recorded by Global Positioning System (GPS) units. 

Specimens were collected from a total of 511 birds belonging to 37 different species. Specifically, 418 wild birds were sampled from the Peloponnese region from January to November of 2022 and 93 from Western Greece from September to November of 2022. The sampling sites of both regions are depicted in Figure 1. Birds were characterized according to their migratory status as residents, passage migrants, summer visitors (breeding), partial migrants (breeding), non-breeding visitors and winter visitors.

### 2.3. Molecular Detection of WNV in Wild Birds

Extraction of viral RNA was performed from blood samples, oropharyngeal swabs and fecal swabs (QIAGEN—QIAamp Viral RNA Mini kit, Hilden, Germany) as well as from 20 mg of brain tissue samples and pooled tissue samples (kidney, heart and liver) (QIAGEN RNeasy Mini kit), according to the manufacturers’ instructions. Particularly regarding swabs, prior to the extraction process they were immersed in 500 μL of PCR grade water, and were shaken and squeezed to the sides of the tube to extract the liquid.

Hexaprimers were used for reverse transcription (RT) by utilizing a commercial cDNA synthesis kit (SuperScript™ First-Strand Synthesis System, Invitrogen, for RT-PCR). A 423 bp fragment of the NS3 region of WNV lineage 2 was amplified by performing nested PCR [28]. PCR products of the second round were visualized by electrophoresis on 2% agarose gel and a 100 bp DNA marker was used to determine their amplicon sizes. The presence of WNV was confirmed on the first eight PCR positive samples, which were selected for Sanger sequencing (3730xl DNA Analyzer, Applied Biosystems, Foster City, CA, USA) due to their early detection, using the primers WN-NS3up2 and WN-NS3do2 of the second PCR round [28]. 

### 2.4. Phylogenetic Analysis

Sequence alignment was performed by using ClustalW, and was based on 398 bases of the NS3 region of 57 WNV strains and one Yellow fever virus (YFV) strain. Specifically, the analysis encompassed eight WNV lineage 2 strains, isolated from Peloponnese Region in 2022 and 49 WNV lineage 1, 2, 3, 4, 5, 7 and 8 strains, retrieved from GenBank database, while YFV (DQ235229.1) was used as an outgroup. The phylogenetic tree was constructed with the Neighbor-Joining algorithm [29] and the evolutionary distances were computed using the LogDet (Tamura–Kumar) method [30]. The rate variation among sites was modeled with a gamma distribution (shape parameter = 0.87). A bootstrap resampling analysis for 1000 replicates was performed to estimate the confidence of tree topologies [31]. Analysis was conducted using MEGA11 software, version 11.0.13 [32]. 

### 2.5. Environmental Variables

Environmental variables (Table 1) comprised climatic conditions, topography, land uses, and human activities. Climate indices were derived from WorldClim version 1.4. [33], digital elevation model was extracted from CGIAR-CSI GeoPortal [34] and hydrological data were retrieved from HydroSHEDS [35]. Human population density and 44 categories of land uses (Table S1) were downloaded from the European Environmental Agency [36,37]. The variable distance from livestock farms (sheep, goats and cattle) was generated for this study from the Geodata base of Laboratory of Microbiology and Parasitology, Veterinary Faculty, University of Thessaly. Eight environmental layers were created for the analysis by using ArcGIS 10·1 GIS software (ESRI, Redlands, CA, USA) and, subsequently, the environmental variables were associated with the presence or absence of WNV RNA in wild birds. Datasets were converted to a common projection map extent and resolution prior to use in the modeling program. 

### 2.6. Statistical Analysis

Statistical analysis was performed using Stata 17 (StataCorp. 2021. Stata: Release 17. Statistical Software. College Station, TX, USA: StataCorp LLC) and evaluated for significance at the 5% level. Descriptive statistics of collected data were performed. The evaluation of the potential association between detection (presence or absence) of WNV RNA in collected samples with the recorded environmental variables was performed with the use of a logistic regression model. Presence or absence of WNV RNA was the dependent variable, while the parameters: (i) annual mean temperature (°C), (ii) maximum temperature of warmest month (°C), (iii) heat stress index, (iv) altitude (m), (v) distance from water collections and hydrographic network (m), (vi) distance from livestock farms (sheep, goats and cattle, m), (vii) land uses and (viii) human population density (people/km^2^) were the independent ones. All independent variables were initially screened one by one in univariate logistic regression models. During this process, a significance level of 0.25 was applied as a screening criterion, since a more traditional level (such as *p* < 0.05) could fail to identify variables known to be important [38]. Subsequently, factors with *p* < 0.25 were offered simultaneously to a full model, successively reduced by backwards elimination [39] until only significant (*p* < 0.05) variables remained. Two-factor interactions between the remaining variables were created and tested for significance, by offering them one at a time to the model. Lastly, previously excluded variables were re-offered one by one to the final model, to ensure that variables that could significantly add to the model were not omitted. 

## 3. Results

### 3.1. Molecular Detection of WNV in Wild Birds

During 2022, a total of 418 wild birds belonging to 39 different species from the Peloponnese region and 93 wild birds from three different species from the Western Greece region were molecularly screened for the presence of WNV. Viral RNA was detected in 71 wild birds of nine species (Table 2, Figure S1). 

In particular, virus presence in the Peloponnese region was demonstrated in 52 birds of nine different species: magpie (n = 37), song thrush (n = 6), hooded crow (n = 2), common starling (n = 2), common blackbird (n = 1), Eurasian jay (n = 1), black-winged stilt (n = 1), great tit (n = 1) and house sparrow (n = 1). Positive samples originated from all five regional units (Argolis n = 12, Arcadia n = 15, Corinthia n = 9, Laconia n = 3 and Messenia n = 13) and were obtained during the months February–April, July and September–November. Sanger sequencing was performed on the eight first strains of the virus obtained from magpies (n = 4), song thrushes (n = 3) and a common starling (n = 1) that were detected as early as the end of February from the regional units of Arcadia, Corinthia and Messenia.

Regarding the Western Greece region, sampled species consisted of magpies, song thrushes and common starling. Nineteen magpies were found positive throughout the sampling period (September to November) and originated from all three regional units (Achaia n = 5, Ilia n = 9 and Aitoloakarnania n = 5).

The virus was detected neither in the birds found dead on the road network (n = 18) nor in the environmental samples (n = 21). 

**Figure 2:**
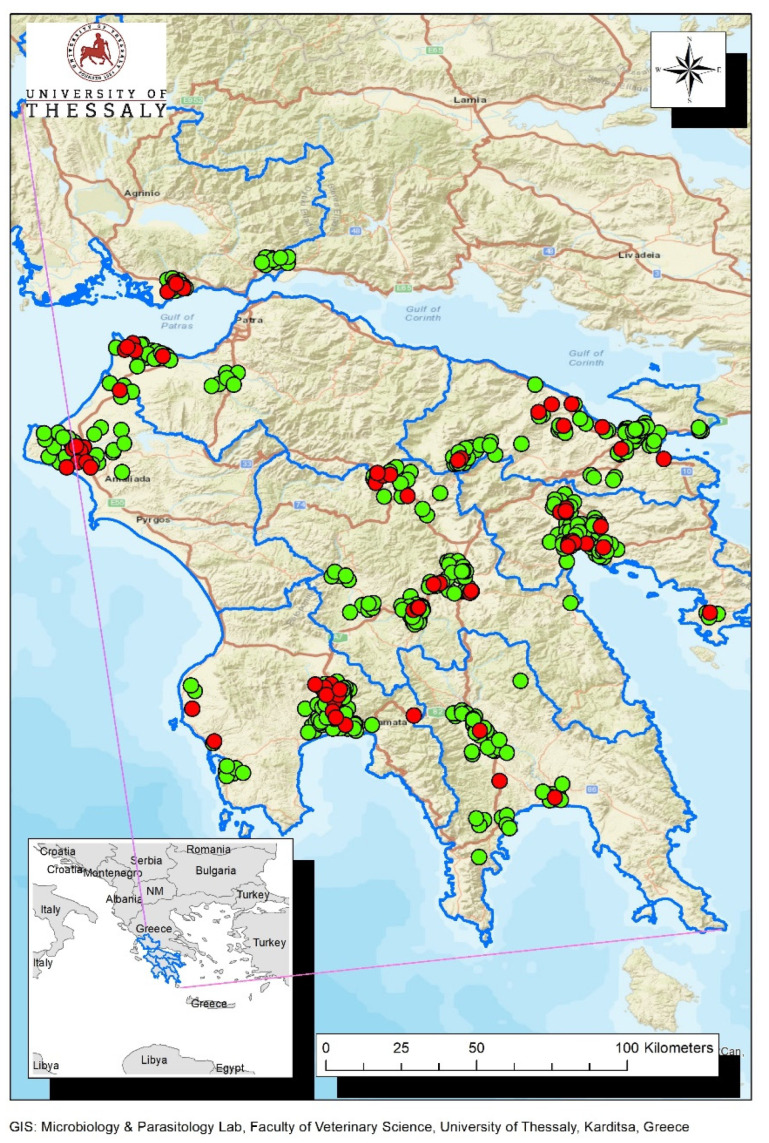
Map of the study area. Locations of WNV positive and negative wild birds are depicted with red and green dots, respectively. The boundaries of the regional units (Peloponnese region: Argolis, Arcadia, Laconia, Corinthia and Messenia, Western Greece region: Achaia, Ilia and Aitoloakarnania) are outlined with blue color.

The results of WNV detection are summarized in Table 2. The sampling sites where positive and negative wild birds occurred are depicted in Figure 2.

### 3.2. Phylogenetic Analysis

**Figure 3:**
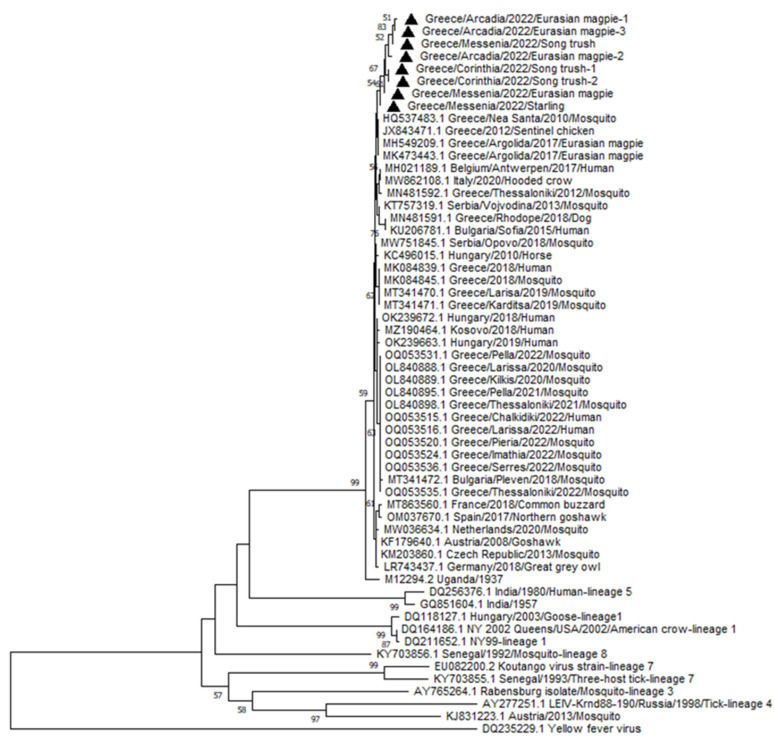
Phylogenetic tree depicting the relationships of eight WNV lineage 2 strains, isolated from the Peloponnese region in 2022 (black triangles), and 49 WNV strains retrieved from GenBank database. YFV strain DQ235229.1 was used as an outgroup. The evolutionary history was inferred using the Neighbor-Joining method and distances were computed using the LogDet (Tamura–Kumar) method. The rate variation among sites was modeled with a gamma distribution (shape parameter = 0.87). Bootstrap values (expressed as percentages of 1000 replications) are shown at the branch points; only values over 50% are indicated. This analysis involved 58 nucleotide sequences and there was a total of 398 positions in the final dataset.

Part of NS3 of the first eight WNV strains was recovered from eight different birds sampled from the geographical areas of Arcadia (n = 3), Corinthia (n = 2) and Messenia (n = 3) of the Peloponnese region during February 2022. Sequences were deposited in GenBank under the accession numbers OR825041-OR825048 (Table S2). According to the phylogenetic tree that was constructed (Figure 3), these strains belong to evolutionary lineage 2 and form a group together, displaying 98.86–100% nucleotide similarity. They show high resemblance (98.46–100%) to those that circulated in Argolis in 2017 and in Macedonia in 2010 and 2012, as well as to Belgian, Italian, Serbian, Bulgarian and Hungarian stains. Peloponnese wild birds’ strains were phylogenetically closer to Greek strains that circulated in Central and Northern Greece prior to 2020 (97.9–99.4%) than to the ones obtained from the same areas after 2020 (97.63–99.7%), as the latter formed a separate group.

### 3.3. Statistical Analysis

After the screening process, two parameters, namely, human population density and distance from water collections and the hydrographic network, were eligible for inclusion in the full model. The final model comprised the aforementioned two variables that were significant and retained. Their interaction was not significant (*p* = 0.453). 

For one unit increase in population density it was 0.996 (OR: 0.996, 95% CI: 0.992; 0.9999, *p* = 0.047) times less likely to obtain a positive PCR result for WNV in bird samples. Practically, one unit increase in population density resulted in a 0.4% (95% CI: 0.01; 0.8) decrease in the odds of obtaining a positive WNV PCR result. Additionally, for one unit increase in distance from water collections and the hydrographic network, it was 0.998 (OR: 0.988, 95% CI: 0.997; 0.999, *p* < 0.001) times less likely to get positive WNV PCR results in bird samples. Essentially, one unit increase in distance from water sources resulted in a 0.2% (95% CI: 0.1; 0.3) decrease in the odds of detecting WNV RNA.

## 4. Discussion

The present study was conducted in the context of an active WNV surveillance program in the Peloponnese and Western Greece regions of Greece during 2022, focusing on the detection of WNV in wild birds. To this end, 511 samples of wild birds were molecularly screened for the virus presence, which was detected in 71.

WNV identification in Peloponnese was evident not only in migratory–winter visitor birds (song thrush, common starling, and black-winged stilt) that could contribute to long distance virus dissemination but also in sedentary birds (magpies, hooded crow, house sparrow, Eurasian jay, and great tit). The engagement of resident birds is considered necessary for the maintenance and the spread of the virus to adjacent regions [42,43]. Surprisingly, WNV circulation season in avian species started up early, as the virus was identified in bird samples in February of 2022. Eight strains were subjected to partial sequencing and the existence of WNV evolutionary lineage 2 was confirmed. According to the phylogenetic analysis, they were grouped together and displayed great similarity to the ones that circulated in Argolis in 2017 and in Macedonia in 2010 and 2012, which were responsible for three major outbreaks [16,21,44]. Hence, our findings indicate potential maintenance of the virus in Peloponnese over the years. During the year 2022, the presence of WNV was also demonstrated in mosquito and human samples from Central and Northern Greece [45], while strains from these areas formed a distinct group, suggesting either a different origin or evolution from the wild bird strains of Peloponnese. Nevertheless, as we performed sequencing in a part of the NS3 WNV genome, analysis on a larger area of the virus genome would provide more reliable information about their relationship. 

Surveillance of WNV in wild birds was implemented for the first time in the Western Greece region, an area neighboring to Peloponnese, during autumn of 2022 (September to November). As the program was initiated with a delay, sampling was focused mainly on magpies, which are a sedentary species that has been shown to present susceptibility to the virus [16]. Additionally, magpies live in proximity to humans in a variety of habitats, and are regarded as an ideal species to be used as sentinels for the surveillance and early detection of WNV both in endemic and non-endemic areas [46]. It shall be noted that, in recent years (2019–2020), WNV has not been detected either among humans or in entomological surveillance studies on female *Cx. pipiens* s.l. mosquitoes that were conducted in Western Greece [18,47]. Thus, our results should be evaluated in subsequent periods of potential virus transmission for prevention of human cases. 

WNV positive birds were detected in Peloponnese as early as in February and until November, and in Western Greece up to November. According to the literature, WNV is usually detected in wild birds between July and September [48], one to two months before human cases, that are most commonly reported during July–October and peak between mid-August and mid-September [49]. Subsequently, our findings evidence a prolonged period of virus circulation in the Peloponnese region, probably due to a seasonal change in the ecology of the virus which could be attributed to a variety of factors. It has been well documented that specific environmental parameters, such as increased ambient temperatures during summer, high precipitation in late winter/early spring or during summer and summer drought, as well as the presence of competent mosquito vectors have been associated with an increase in the virus’ circulation [50,51,52,53]. A possible overwintering of the virus in the population of wild birds could be assumed, as long-term persistence of WNV in infected animals may result in the infection of birds by prey ingestion even months after the end of the mosquito season, providing a mechanism of overwintering [54,55]. Recent studies from other European countries have also supported the local overwintering of WNV in wild birds in Spain [56] or mosquitoes in Germany and the Czech Republic [57,58]. Interestingly, Montecino-Latorre D. and Barker C.M (2018) constructed a dynamical model to simulate a bird community consisting of crows, raptors and other birds in order to evaluate whether WNV could persist through winter under plausible bird-to-bird transmission parameters while causing realistic outbreaks in the absence of mosquito-borne transmission [59]. The study concluded that birds can sustain WNV through winter via bird-to-bird transmission, specifically due to a limited but persistent number of new WNV crow-to-crow transmission events [59]. Another potential scenario for our results is that the virus could have spread southwards during the autumn migration of birds from Northern Europe to wintering areas in Africa with resting stops in Greece. This hypothesis has previously been supported with the detection of WNV antibodies in young migratory turtle doves born in northern Europe, on their arrival in Greek territory [15]. 

To get a better understanding of the virus’ ecology, the locations of positive and negative birds for WNV RNA were associated with eight environmental variables. Two parameters, namely human population density and distance from water collections and the hydrographic network, were statistically significant. It was more likely to identify a PCR positive bird as the population density decreased. This was expected considering that WNV is maintained in nature in a mosquito–bird cycle and wild birds mainly inhabit rural ecosystems, while their presence in the vicinity of humans is mainly driven by their need to feed. Additionally, a decrease in the distance from water sources resulted in an increase in the odds for WNV detection among the tested wild birds. Wetlands are the most important ecosystems for enzootic transmission of the virus due to the presence of both resident and migratory bird as well as of competent mosquito vectors, particularly *Cx*. *pipiens* [60]. Overall, water bodies are considered to favor the activity of mosquitoes and their capacity to complete different life cycle stages [61]. As such, short distances from water bodies have been shown to be associated with more WNV human and animal cases, mainly due to increased mosquito exposure [62]. This information, that associates the virus’ ecology with human population density and distance from water collections, could be particularly useful for future surveillance programs in the country, as it could be utilized to adjust the sampling locations of wild birds. Targeted surveillance of wild birds and vector control efforts prioritized for high WNV risk areas could lead to increased public health protection during outbreaks while reducing costs, labor and environmental impacts. 

During 2022, 286 human cases of WNV were recorded in five regions of Greece, while the virus was also identified in human and mosquito samples [18,45]. In our study, WNV was molecularly detected in wild bird species, and especially in magpies in both Peloponnese and Western Greece. Given that positive birds were identified as early as in late February and until as late as in November, it is suggested that surveillance of avian species should be imposed throughout the year. Ideally, magpies could be used as sentinels for WNV monitoring, since identification of virus spread among them could lead in the implementation of preventive measures that would hamper disease outbreaks in humans. The implementation of an integrated approach in Greece, involving human health, animal health and vector control strategies is proposed as the most efficient and effective mechanism for tackling WNV transmission. 

## 5. Conclusions

This study provides evidence of WNV lineage 2 circulation among different wild bird species in the regions of Peloponnese and Western Greece, during 2022, and highlights the possibility of the virus’ overwintering. The presence of WNV among wild birds was associated with decreased population density and decreased distance from water sources, a finding that could be implemented in future surveillance programs in the country.

## Figures and Tables

**Table 1:** Environmental variables.

Environmental Variable	Code
Annual mean temperature (°C)	clima1
Maximum temperature of warmest month (°C)	clima2
Heat stress index ^1^	HI
Altitude (m)	dem
Distance from water collections and hydrographic network (m)	waterdis
Distance from livestock farms (sheep, goats and cattle, m)	farmdis
Land uses (44 classes)	landcorine
Human population density (people/km^2^)	popden

^1^ Heat stress index: number of days that experienced ‘strong heat stress’ (UTCI between 32 and 38 °C) during June, July and August 2022. (Data source: ERA5-HEAT. Credit: C3S/ECMWF.)

**Table 2:** Tested and WNV positive wild bird species from the Peloponnese and Western Greece regions during 2022.

Scientific Name	Common Name	Status ^a^	Regional Units of Peloponnese Region					Regional Units of Western Greece Region		
			Argolis	Arcadia	Corinthia	Laconia	Messenia	Achaia	Ilia	Aitoloakarnania
*Anas (Mareca) penelope*	Eurasian wigeon	WV, PM	0/1	-	-	-	-	-	-	-
*Anas platyrhynchos*	Mallard	WV, r	0/1	-	-	-	-	-	-	-
*Ardea cinerea*	Grey heron	R, PM	0/2 ^Ε^	-	-	-	-	-	-	-
*Ardeola ralloides*	Squacco heron	SV, PM	0/1 ^Ε^	-	-	-	-	-	-	-
*Buteo buteo*	Common buzzard	R, WV	0/1 ^D^	0/1 ^D^	-	-	-	-	-	-
*Calidris* spp.	-	PM, WV	0/3 ^E^	-	-	-	-	-	-	-
*Carduelis carduelis*	European goldfinch	R, wv	0/1	-	-	-	0/2	-	-	-
*Cettia cetti*	Cetti’s warbler	R	-	-	-	-	0/2	-	-	-
*Charadius dubius*	Little ringed plover	SV, PM	0/3 ^E^	-	-	-	-	-	-	-
*Columba livia*	Rock dove	R	0/1	-	-	-	0/4 + 1 ^D^	-	-	-
*Corvus cornix*	Hooded crow	R	0/2	0/5	0/11	1/17	1/7	-	-	-
*Corvus (Coloeus) monedula*	Western jackdaw	R	-	0/6	-	-	-	-	-	-
*Egretta garzetta*	Little egret	PM, R	0/3 ^E^	-	-	-	-	-	-	-
*Erithacus rubecula*	European robin	WV, r	0/2	0/1 + 1 ^D^	0/1	-	0/1	-	-	-
*Fringilla coelebs*	Common chaffinch	R, WV	-	0/1 ^D^	-	-	0/1 ^D^	-	-	-
*Gallinago gallinago*	Common snipe	WV, PM	0/1	-	-	-	-	-	-	-
*Garrulus glandarius*	Eurasian jay	R	0/6	0/3	1/1	-	0/3	-	-	-
*Himantopus himantopus*	Black-winged stilt	PM, SV	1/6	-	-	-	-	-	-	-
*Hirundo rustica*	Barn swallow	SV, PM	-	-	0/1 ^D^	-	-	-	-	-
*Induna (Iduna) pallida*	Eastern olivaceous warbler	SV	-	-	-	0/3	0/4	-	-	-
*Lanius collurio*	Red-backed shrike	SV, PM	-	0/1	-	-	-	-	-	-
*Larus michahellis*	Yellow-legged gull	R	0/2 ^E^ + 1 ^D^	-	-	-	-	-	-	-
*Larus (Chroicocephalus) ridibundus*	Black-headed gull	WV, r	0/5 ^E^	-	0/3	-	-	-	-	-
*Numerius (Numenius) arquata*	Eurasian curlew	WV, PM	0/1 ^E^	-	-	-	-	-	-	-
*Otus scops*	Eurasian scops owl	PLM	0/1^D^	-	0/1	-	-	-	-	-
*Parus major*	Great tit	R	-	-	-	1/4	0/3	-	-	-
*Passer domesticus*	House sparrow	R	0/4	0/7	0/13 + 1 ^D^	0/2	1/6	-	-	-
*Phalacrocorax carbo*	Great cormorant	WV, r	0/1 ^E^	-	-	-	-	-	-	-
*Pica pica*	Magpie	R	9/47 + 2 ^D^	15/58 + 2 ^D^	4/36	0/4	9/37	5/22 + 1 ^D^	9/38	5/14
*Scolopax rusticola*	Eurasian woodcock	WV, r	-	-	0/2	0/1	-	-	-	-
*Streptopelia decaocto*	Eurasian collared dove	R	-	-	0/8	-	-	-	-	-
*Strix aluco*	Tawny owl	R	-	-	0/2 ^D^	-	-	-	-	-
*Sturnus vulgaris*	Common starling	WV, R	-	0/1	1/1	-	1/3	0/8	-	0/7
*Sylvia (Curruca) communis*	Common whitethroat	SV, PM	-	0/1	-	-	-	-	-	-
*Sylvia (Curruca) melanocephala*	Sardinian warbler	R	-	-	0/2	0/1	0/2	-	-	-
*Turdus merula*	Common blackbird	R, WV	-	-	0/1	1/4	0/1	-	-	-
*Turdus philomelos*	Song thrush	WV, r	2/8	0/4	3/10	0/8 + 1 ^D^	1/4	-	-	0/3

R—resident, PM—passage migrant, SV—summer visitor (breeding), PLM—partial migrant (breeding), WV—winter visitor; -—no samples were tested; ^a^ Capital letters denote that the species is common in this category while small letters that it is rare [40,41]; D—dead wild birds; E—environmental samples.

## Data Availability

All data generated for this study are presented within the manuscript and the supplementary files and are available upon reasonable request from the corresponding author.

## Supplementary Materials

The following supporting information can be downloaded at: https://www.mdpi.com/article/10.3390/vetsci10110661/s1, Figure S1: Gel electrophoresis of WNV positive strains; Table S1: Categories of land uses; Table S2: Sequences were deposited in GenBank.
