# Supplementary figures and images for: Indication of West Nile Virus (WNV) Lineage 2 Overwintering among Wild Birds in the Regions of Peloponnese and Western Greece
Source: Vet Sci. 2023 Nov 18;10(11):661. doi: 10.3390/vetsci10110661 (PMC10674244; doi:10.3390/vetsci10110661)

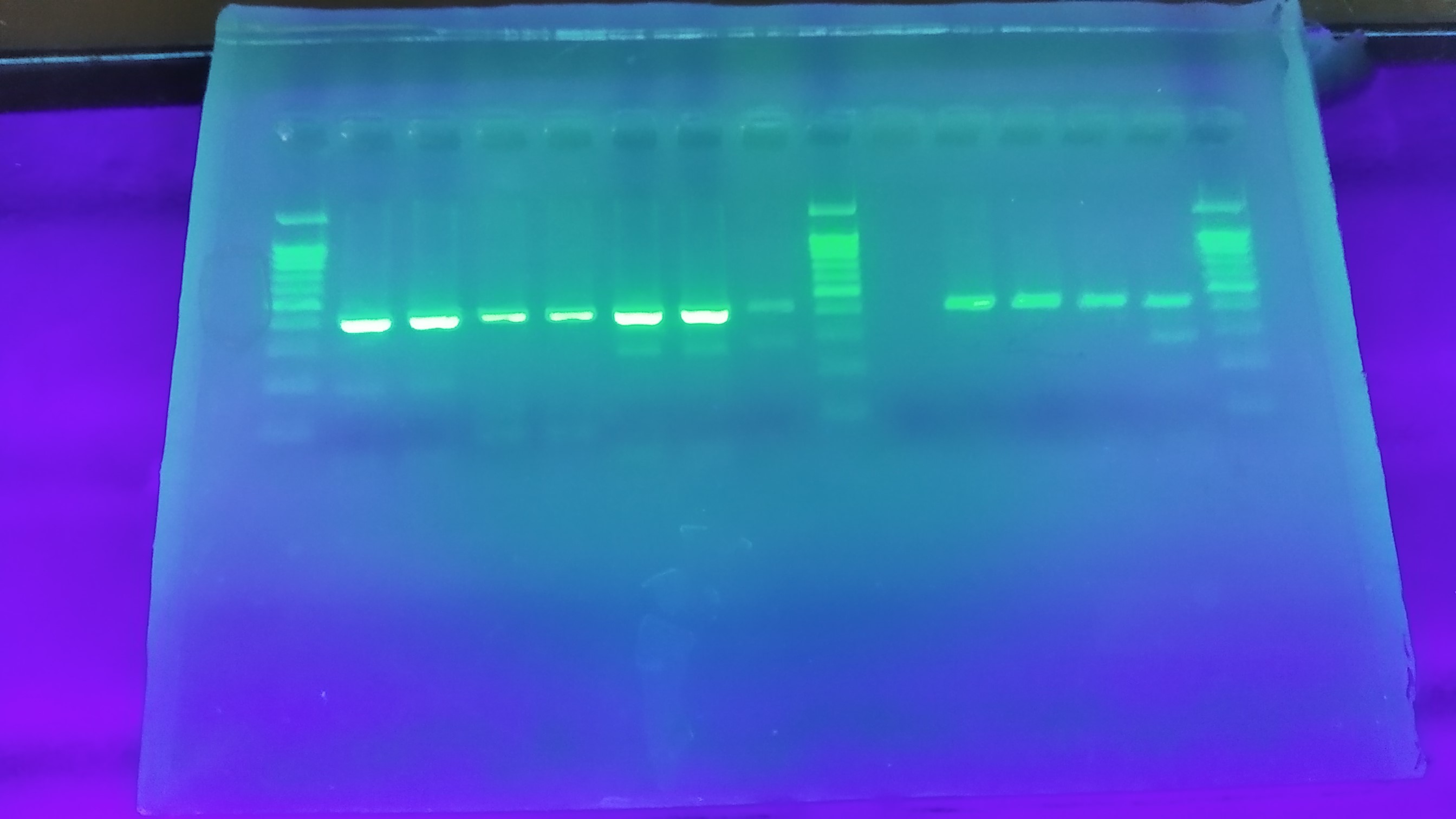

Supplement: Supplementary file 1 [file vetsci-10-00661-s001.zip › Figure S1.jpg]
